# CLIMBra - Climate Change Dataset for Brazil

**DOI:** 10.1038/s41597-023-01956-z

**Published:** 2023-01-20

**Authors:** André Simões Ballarin, Jullian Souza Sone, Gabriela Chiquito Gesualdo, Dimaghi Schwamback, Alan Reis, André Almagro, Edson Cezar Wendland

**Affiliations:** 1 Department of Hydraulics and Sanitation, São Carlos School of Engineering, University of São Paulo, CxP. 359, São Carlos, São Paulo, 13566-590 Brazil; 2 Faculty of Engineering, Architecture and Urbanism, and Geography, Federal University of Mato Grosso Do Sul, CxP 549, Campo Grande, Mato Grosso Do Sul 79070-900 Brazil

**Keywords:** Projection and prediction, Hydrology, Climate-change impacts

## Abstract

General Circulation and Earth System Models are the most advanced tools for investigating climate responses to future scenarios of greenhouse gas emissions, playing the role of projecting the climate throughout the century. Nevertheless, climate projections are model-dependent and may show systematic biases, requiring a bias correction for any further application. Here, we provide a dataset based on an ensemble of 19 bias-corrected CMIP6 climate models projections for the Brazilian territory based on the SSP2-4.5 and SSP5-8.5 scenarios. We used the Quantile Delta Mapping approach to bias-correct daily time-series of precipitation, maximum and minimum temperature, solar net radiation, near-surface wind speed, and relative humidity. The bias-corrected dataset is available for both historical (1980–2013) and future (2015–2100) simulations at a 0.25° × 0.25° spatial resolution. Besides the gridded product, we provide area-averaged projections for 735 catchments included in the Catchments Attributes for Brazil (CABra) dataset. The dataset provides important variables commonly used in environmental and hydroclimatological studies, paving the way for the development of high-quality research on climate change impacts in Brazil.

## Background & Summary

General Circulation and Earth System Models (GCMs/ESMs) play an important role in simulating the physics and dynamics of the Earth system, as well as in assessing and understanding projected changes in the global climate^1,2^. The employment of climate models along with observed meteorological data is key to inform policy and decision-making based on hydroclimatic modeling^3–6^. Nevertheless, to provide a large volume of climatic data at a global scale, GCMs/ESMs often present (i) coarse spatial resolution (100–300 km), hampering the development of reliable and detailed studies at finer scales^7^ and (ii) systematic biases, leading to misrepresentation of different statistical properties of observed climate variables^8,9^. Apart from these two limitations, these climate models have intrinsic uncertainties that undermine studies on climate change impacts^10^. In this context, statistical and dynamical downscaling approaches are used to bridge the inherent gap between projected and observed data by improving the spatial resolution of GCMs products to a finer scale. Dynamical downscaling is based on the integration of Regional Climate Models (RCMs) — which are able to capture local features and dynamics to better represent the climate of a specific and limited area — with the initial and lateral boundary conditions derived from GCMs^11,12^. Despite the improvement in GCMs resolution and the incorporation of local scale-effects into their projections, RCMs may still contain systematic biases that are propagated through the future simulations. These remaining biases, derived from the used GCMs or incorporated during the dynamical downscaling procedure due to limited understanding of the processes, may lead to the need of further corrections of the dynamically downscaled product^13–15^ at a large computational effort^16^. On the other hand, the statistical downscaling is based on statistical transfer functions that adjust the probability distribution function of projections to resemble observed data at local/regional sites^17^. The statistical framework is able to correct systematic biases found in the original projections of both RCMs (generally referred to as “hybrid downscaling”^18,19^) and GCMs using simple modelling structures that require less computational effort^20,21^. Given these advantages, the statistical approach is generally preferred over the dynamical for climate-based studies^3,22^.

Despite the importance of a dataset with historical and future data for meteorological and hydrological studies, to our knowledge there is no dataset of bias-corrected climate change data available for the Brazilian territory based on the recently released Sixth Assessment Report (AR6) of the Coupled Model Intercomparison Project phase 6 (CMIP6). Brazil is a continental country with diverse hydroclimatic conditions, and climate-related hazards have become more frequent, widespread, and interconnected^23–28^. Thereby, here we developed the Climate Change Dataset for Brazil - CLIMBra, a bias-corrected dataset comprising six important meteorological variables used in hydro-climatic and economic studies related to climate change: precipitation (*pr*), maximum (*tasmax*) and minimum temperature (*tasmin*), net shortwave surface radiation (*rss*), near-surface wind speed (*sfcWind*) and relative humidity (*hur*). Besides the bias-corrected gridded daily data at a spatial resolution of 0.25°, we provide an area-averaged, point-based scale data for 735 catchments of the Catchments Attribute for Brazil (CABra) large-sample dataset^29^. The developed dataset consists of bias-corrected historical (1980–2013) and future (2015–2100) simulations of 19 GCMs/ESMs, forced by the CMIP6 SSP2-4.5 and SSP5-8.5 scenarios. Our product is of paramount importance to support climate change assessment in the context of the water-energy-food nexus and hence inform policy and decision-making.

## Methods

### Datasets

To generate the bias-corrected product, we used observed data covering the historical period (1980–2013) and simulated data covering both historical and future (2015–2100) periods. As observed data, we adopted the meteorological dataset developed by Xavier *et al*.^30^, which includes gridded daily series with 0.25° × 0.25° spatial resolution for the six meteorological variables evaluated in this study. This dataset uses data from 3,625 ground-based rain gauges and 735 weather stations provided by the National Institute of Meteorology (INMET), National Water and Sanitation Agency (ANA), and the Water and Electric Power Department of São Paulo (DAEE/SP) as input to produce interpolations. Six different interpolation techniques were evaluated: arithmetic averaging, thin plate spline, natural neighbor, inverse distance weighting, angular distance weighting, and ordinary point kriging. In general, the dataset shows a good performance in describing the weather station observations and is widely applied in hydrological and climatological studies in Brazil^31,32^, and also as the ground truth to GCMs/RCMs projections assessment and impact studies^33,34^.

For historical and future projections, we used daily data from 19 CMIP6 GCMs\ESMs (Table 1). The models were selected based on the following criteria: (1) availability of daily data for the evaluated climate variables under the r1i1p1f1 variant, and (2) nominal spatial resolution up to 250 km. For relative humidity (*hur*), wind speed (*sfcWind*), and net solar radiation (*rss*), only 10 GCMs were available at the stipulated conditions. For future projected changes, we considered two Shared Socioeconomic Pathways: the middle of the road (SSP2-4.5) and the fossil-fueled development (SSP5-8.5). The latter scenario represents the high end of future pathways with enough emissions to achieve a radiative forcing of 8.5 W.m^−2^ by 2100, whilst SSP2-4.5 represents the medium part of the range of future pathways. The two scenarios are an update of the previous Representative Concentration Pathways (RCPs) from the CMIP5^35,36^. The SSPs include mitigation and adaptation efforts based on economic and social changes, such as societal-economic development^37^. There are two underlying reasons behind considering these scenarios: (1) since they represent the intermediate (SSP2-4.5) and extreme (SSP5-8.5) future climate change conditions, they are able to represent a wide range of expected changes in global climate dynamics, encompassing other scenarios, such as the SSP3-7.0, which were not available for all evaluated CMIP6 GCMs/ESMs at the time of the pre and post-processing tasks; (2) they are the most used future scenarios in Brazilian climate change studies^12,33,34,38^. The main climatological institute of the country, the National Institute for Space Research (INPE), provides RCM-simulated future data for the country considering the two forcing-equivalent CMIP5 RCPs scenarios (RCP4.5 and RCP8.5)^11^. Therefore, the use of these scenarios here may enable future CMIP5-CMIP6 comparison studies.

**Table 1:** CMIP6-GCMs\ESMs used in our dataset.

Model	Country/Region	Resolution	Reference
MRI-ESM2*	Japan	1.12° × 1.12°	^60^
EC-EARTH3*	Europe	0.7° × 0.7°	^61^
CMCC-ESM2*	Europe	0.9° × 1.25°	^62^
INM-CM4-8*	Russia	1.5° × 2.0°	^63^
NorESM2-MM*	Norway	0.9° × 1.25°	^64^
MPI-ESM1.2-HR*	Germany	0.9° × 0.9°	^65,66^
INM-CM5*	Russia	1.5° × 2.0°	^67^
ACCESS-ESM1-5*	Australia	1.87° × 1.25°	^68,69^
TaiESM1	Taiwan	1.9° × 1.25°	^70^
NESM3	China	1.9° × 1.9°	^71^
KIOST-ESM	South Korea	1.87° × 1.87°	^72^
K-ACE	South Korea	1.87° × 1.25°	^73^
GFDL-CM4	USA	1.0° × 1.25°	^74^
GFDL-ESM4	USA	1.0° × 1.25°	^75^
ACCESS-CM2	Australia	1.87° × 1.25°	^76^
HadGEM3-GC31-LL	UK	1.87° × 1.25°	^77^
IPSL-CM6A*	France	2.5° × 1.3°	^78^
UKESM1.0	UK	1.87° × 1.25°	^79^
MIROC6*	Japan	1.4° × 1.4°	^80^

^*^Simulations available for all evaluated variables.

### Pre and post-processing

Given the coarse and different spatial resolutions of the CMIP6 GCMs/ESMs, we performed a bilinear interpolation following previous studies (e.g.^3,6,10,39^) to regrid all models to a common 0.25° spatial grid. Moreover, to obtain a multi-model ensemble seeking to encompass different representations and uncertainties of all evaluated models, the GCMs outputs need to be in the same spatial grid resolution^40,41^. It is worth mention that regridding may severely impact the statistical properties of meteorological variables, especially those linked to extreme events^42^. Hence, such limitation should be considered in future studies using our dataset.

Besides the coarse spatial resolution exhibited by GCM/ESMs, they also show an inherent inability to simulate the present-day climate conditions leading to systematic errors that are propagated for future simulations^43^. Thus, climate products often require bias correction. Here, we used the Quantile Delta Mapping (QDM) approach^20^ since it explicitly preserves relative or absolute changes in quantiles between historical and future simulations^44^. This method is based on two widely used correction procedures: the quantile delta change and the detrend quantile mapping^45,46^. According to Cannon *et al*.^20^, the QDM can be performed in three steps. First, all the individual future projected quantiles are detrended. Then, the detrended quantiles are bias-corrected using the quantile mapping technique. Lastly, the projected changes are then superimposed on the bias-corrected outputs. Let denote *o* and *p* as observed and projected data, and *h* and *f* as historical and future periods, respectively. The definition of the non-exceedance probability of observed (*x*_*h,o*_) and projected historical (*x*_*h,p*_) data, and future data (*x*_*f,p*_) are accounted as:

1 $${p}_{f,p}(t)=F\left({x}_{f,p}(t)\right)$$

$${p}_{h,p}(t)=F\left({x}_{h,p}(t)\right)$$

$${p}_{h,o}(t)=F\left({x}_{h,o}(t)\right)$$

where *p* and *F* respectively denote the non-exceedance probability associated with a specific value at time *t* and the empirical cumulative distribution function (ECDF). We adopted the non-parametric probability distributions as they showed better performance over the parametric ones when downscaling both RCMs and GCMs\ESMs outputs^17^. Also, it is easier to apply it to different meteorological variables, despite their different underlying distributions^3^. Then, we computed a change factor (Eq. 2), which associates the historical simulation output with that of the future period:

2 $${\Delta }^{M}(t)=\frac{{F}_{f,p}^{-1}\left({p}_{f,p}(t)\right)}{{F}_{h,p}^{-1}\left({p}_{f,p}(t)\right)}=\frac{{x}_{f,p}(t)}{{F}_{h,p}^{-1}\left({p}_{f,p}(t)\right)}$$

$${\Delta }^{A}(t)={F}_{f,p}^{-1}\left({p}_{f,p}(t)\right)-{F}_{h,p}^{-1}\left({p}_{f,p}(t)\right)={x}_{f,p}(t)-{F}_{h,p}^{-1}\left({p}_{f,p}(t)\right)$$

where *F*^−1^ indicates the inverse ECDF; and Δ^*M*^(*t*) and Δ^*A*^(*t*) are respectively the multiplicative and the additive change factor between simulated quantiles of the historical and future periods. The former is suitable for precipitation variables preserving relative changes between quantiles, whereas the latter preserves absolute changes in projected quantiles, suitable for temperature-derived variables^20,44^. For precipitation, a frequency adaptation suggested by Themeßl *et al*.^47^ was also applied in order to account for a methodological problem that arises when the frequency of modelled dry days is greater than the frequency of observed dry days, resulting in a systematic wet bias. Moreover, we used a wet-day threshold of 1 mm/day following previous studies^3,17,48^ to minimize drizzle effects.

The bias-corrected projected data are computed according to Eq. 3, following the (i) quantile-mapping technique, which statistically transforms the distribution of the projected data to resemble the distribution of the observed data; and (ii) the change factor approach, which superimposes relative (or absolute) changes between historical and future projected values:

3 $${\widehat{x}}_{f,p}(t){=\Delta }^{M}(t).{F}_{h,o}^{-1}\left({p}_{f,p}(t)\right)$$

$${\widehat{x}}_{f,p}(t){=\Delta }^{A}(t)+{F}_{h,o}^{-1}\left({p}_{f,p}(t)\right)$$

**Fig. 1:**
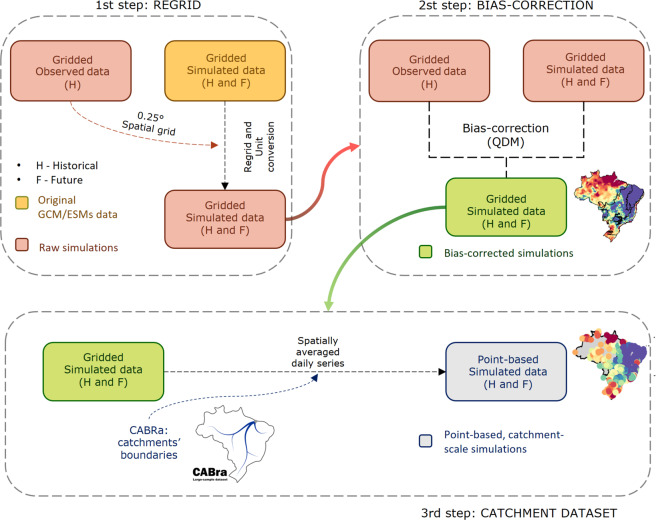
Flowchart representing the core steps used to generate the CLIMBra’s products. Step 1 and 2 represent the regrid and bias-correction tasks, respectively. Step 3 represents the framework required to rescaled the gridded dataset to the CABra’s catchments.

We considered the entire observed period (1980–2013) to correct the future projections (2015–2100) with the QDM algorithm. Both pre and post-processing tasks, depicted in Fig. 1, were carried out using the *downscaleR* package, which is an R-based framework developed to address the needs of different climate impact studies within the *Climate4R* project^18,49^. Despite the wide range of applications of QDM in climate change impact assessments, we must point out the main limitations and uncertainties related to its usage. In general, the QDM (1) shows high sensitivity to the historical reference data used for calibration and (2) may be affected by the downscaling process due to a resolution mismatch between the model simulations and observations^50^. To reduce this uncertainty, high-quality reference data are required. Therefore, we used a high-resolution meteorological gridded dataset^30^ that comprises the largest number of ground-based observations in Brazil. We also highlight our effort to mitigate the introduction of more uncertainty and overcome some methodological limitations for the development of a reliable product for climate change assessment in this country. Nevertheless, it is known that the use of bias-correction methods is controversial^51^ as they are not able to retain spatial and intervariable dependencies^52^. Lastly, bias-corrections methods may produce physically unrealistic values and also hind some fundamental models deficiencies^50,51^.

### Catchment-scale dataset

**Fig. 2:**
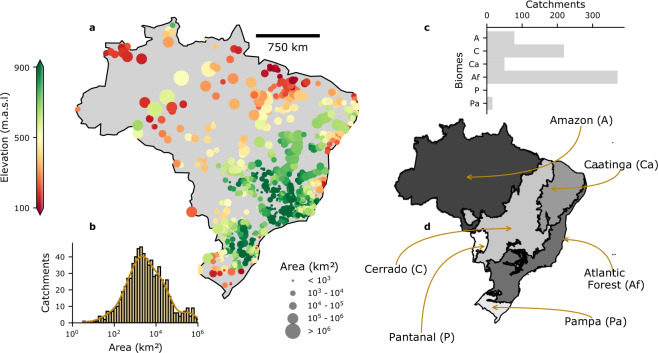
(**a**) Streamflow gauge coordinates of CABra’s catchments, colored according to their mean elevation and sized by their area. (**b**) Histogram of catchments’ area. (**c**) Distribution of catchments per Brazilian biome. (**d**) Six main Brazilian biomes.

The development of a regional gridded meteorological dataset aims at advancing hydro-climate studies in Brazil. Nevertheless, working with gridded climate variables is not a trivial task, often requiring high computation effort. Therefore, we also developed a catchment-scale version of the dataset in order to assist climate-change impact studies/applications (Fig. 1) for both scientific and technical fields. To this end, we rescaled our gridded dataset to match the catchments in the CABra large-sample dataset^29^. CABra includes a set of more than 100 observed climate, hydrological, and physiographic attributes for 735 Brazilian catchments (Fig. 2).

For each CABra’s catchment, we generated a gridded meteorological daily series comprising its extension. In sequence, we spatially averaged the grid-cell time series within the catchment boundaries. This process was conducted for each variable and climate change scenario. Given the continental extensions of Brazil, the country shows a large range of catchment areas. Thus, for some catchments, the daily time series were obtained using only one grid and, for others, averaging more than 50 grids. Moreover, our gridded dataset only comprises the Brazilian territory, and hence the averaged daily time-series only accounted for the catchments’ area within the country boundaries. To compute the spatial averaged daily time series, we weighted the grids according to their latitude, as, for regular grids, the grid cell’s area changes as you move towards the poles. It is also worth noting that the averaged time-series may hinder extreme events in large catchments since extreme high or low events recorded in a specific grid may be smoothed by non-extreme events recorded in neighboring grids when taking the average.

## Data Records

The present study describes a gridded dataset (0.25° × 0.25°) and a spatially averaged dataset at a catchment-scale. The first includes raw and bias-corrected gridded (*netCDF*) daily time-series (*pr*, *tasmax*, *tasmin*, rss*, sfcWind*, and hur*) of 19 (10*) CMIP6 GCMs/ESMs (Table 1) for both the historical (1980–2013) and future (2015–2100) periods. For the future period, two CMIP6 SSP-scenarios were considered: SSP2-4.5 and SSP5-8.5. The second part of the dataset consists of a point-based (*.csv*) daily time-series derived from the aforementioned bias-corrected gridded dataset for 735 Brazilian catchments in the CABra dataset^29^. The time-series from this dataset were also generated considering the two SSP scenarios simulated by the 19 (10) CMIP6 GCMs\ESMs. Both datasets are freely available (CC0 license) at Ballarin *et al*.^53^.

## Technical Validation

### Bias-correction performance

#### Mean and extreme values

**Fig. 3:**
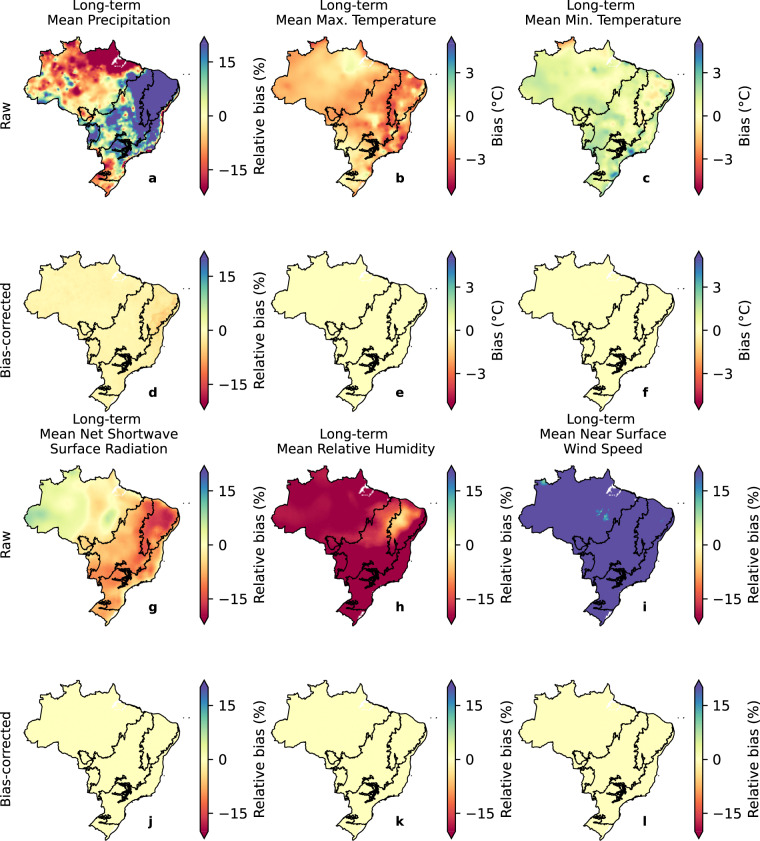
Biases in long-term mean precipitation, maximum and minimum temperature, net shortwave surface radiation, relative humidity, and near surface wind speed considering the gridded dataset in both raw and bias-corrected conditions. The limits of Brazilian biomes are indicated in black borderlines.

To get a preliminary overview of the bias-correction performance in describing the observed data, we computed the relative bias between the estimated and observed long-term means for the historical period (Fig. 3). We used absolute bias for the variables *tasmax* and *tasmin* since the denominator approaches to zero in many catchments, resulting in very high relative bias values. For both raw and bias corrected datasets, we estimated the long-term mean for each individual model and then computed the multi-model ensemble. We repeated this process for all validation analyses of the study. The bias correction significantly improved the performance of the historical simulations in describing observed long-term mean values for all the meteorological variables by reducing the bias to approximately zero.

We found the largest bias for *sfcWind* and *hur* when analyzing the ensemble data prior to bias correction. For *sfcWind*, overestimations of more than 30% were observed throughout the country. The same was noted in *hur*, but with the opposite signal. In general, the raw simulations were unable to capture long-term mean maximum and minimum temperatures. Similar to the findings of Mishra *et al*.^3^, the raw multi-model ensemble exhibited an overall cold bias (−1.70 °C) for *tasmax* and a warm bias (0.87 °C) for *tasmin*. Regarding the long-term mean precipitation, we observed an overall wet relative bias over the country (11.74%), except in the Amazon and Pampa biomes (northern and southern of Brazil, respectively) where we noted a relative dry bias. In general, the CMIP6 models exhibited good performance in estimating the long-term mean *rss*, showing a smaller mean bias (−4.34%) compared with the deviations found for the other variables.

**Fig. 4:**
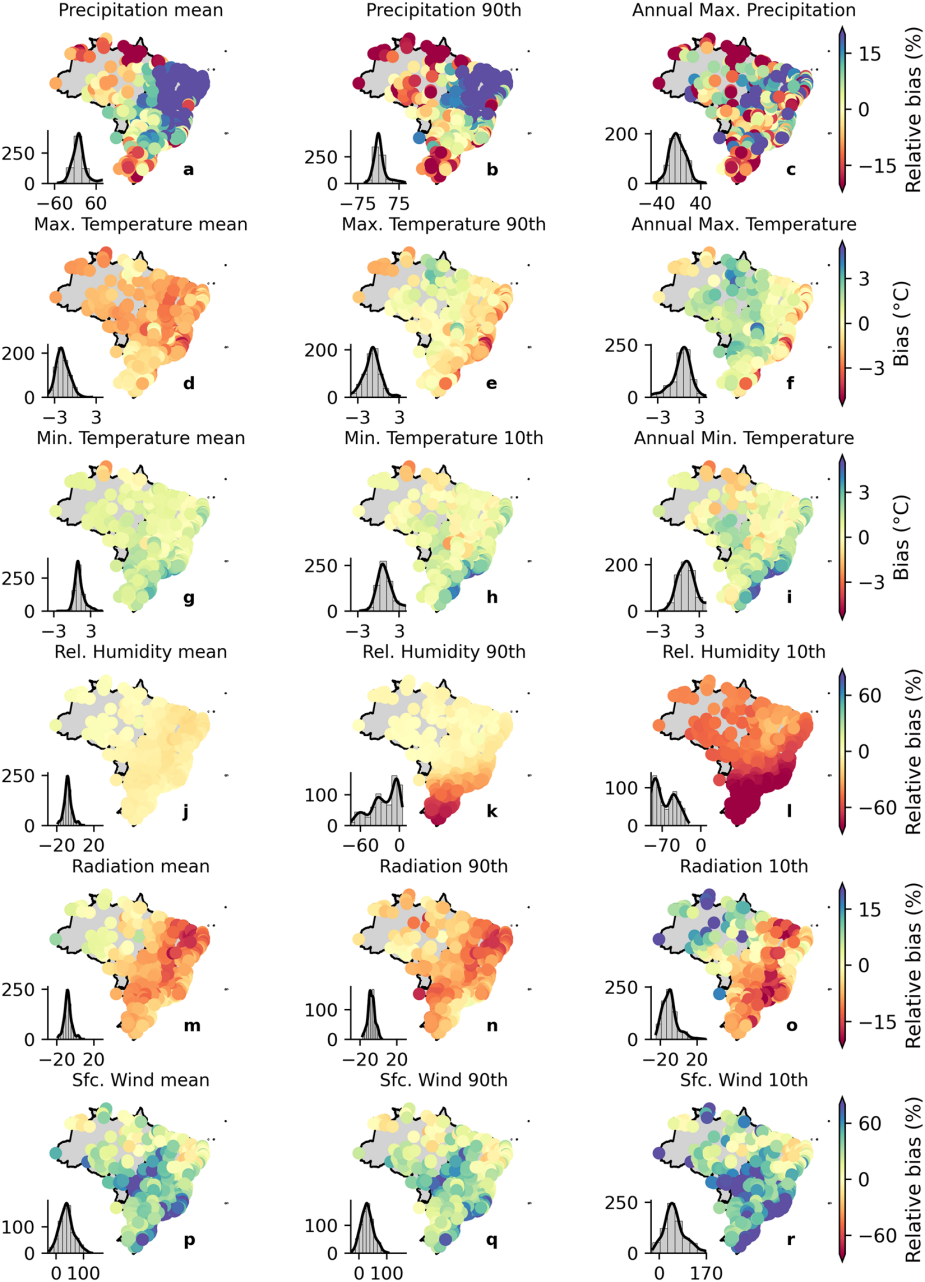
Biases in the long-term mean and extreme values of precipitation, maximum and minimum temperature, net shortwave surface radiation, relative humidity, and near surface wind speed (catchment-scale dataset) for the raw simulations. Histograms in each of the panels indicate the frequency of occurrence of bias.

**Fig. 5:**
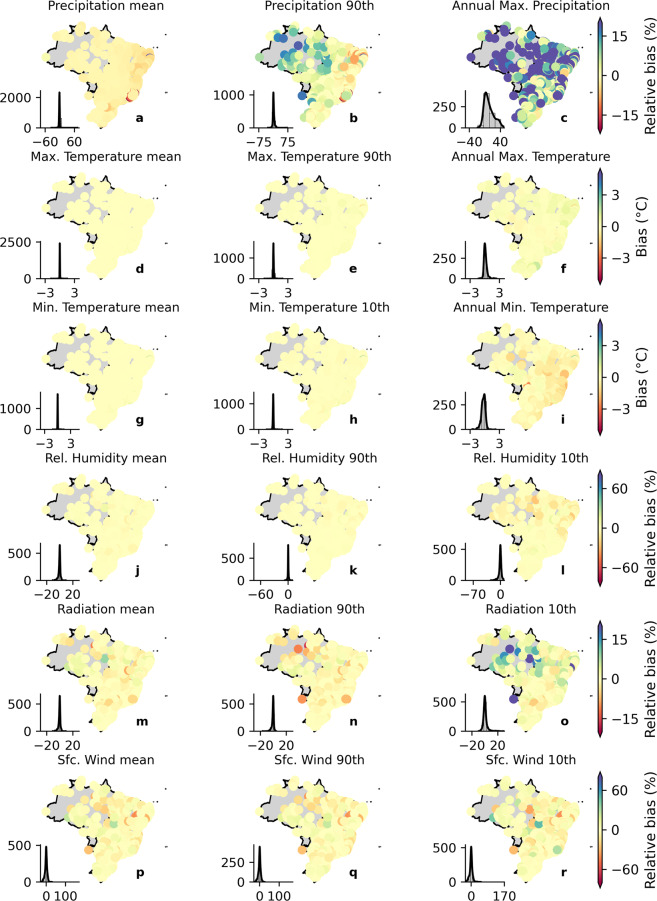
Biases in the long-term mean and extreme values of precipitation, maximum and minimum temperature, net shortwave surface radiation, relative humidity, and near surface wind speed (catchment-scale dataset) for the bias-corrected simulations. Histograms in each of the panels indicate the frequency of occurrence of bias.

In the remainder of this section, we discuss the dataset’s performance considering our catchment-scale product, which is the main product of this study, since it provides ready-for-use meteorological time-series required for most hydrological studies and climate change impacts simulation. Nevertheless, similar conclusions can be drawn for the gridded dataset, which was the basis for the development of the catchment-scale product. To explore the models’ performance beyond the characterization of mean values, we also computed the bias in the variables’ long-term extreme properties: 90th (and/or 10th) percentiles and maximum (and/or minimum) records (Figs. 4, 5). It is worth mentioning that the spatial distribution of the bias in the long-term mean for the gridded (Fig. 3) and catchment-scale datasets is alike (the first column in Figs. 4, 5, plots *a*, *d*, and *g*), corroborating their equivalence in terms of performance. That is, although we spatially averaged the gridded dataset, the catchment-scale product is able to maintain the spatial distribution of relative bias.

In general, the CMIP6 models exhibited poor performance in simulating extreme values. The raw multi-model ensemble showed an overall warm bias (overestimation) for the long-term 10th percentile of *tasmin* and a cold bias (underestimation) for the long term 90th percentile of *tasmax*. For the long-term maximum *tasmax* and minimum *tasmin*, we found an overall warm bias with a more heterogeneous spatial distribution than those observed in the long-term extreme percentiles. Interestingly, *tasmax* and *tasmin* showed a contrasting spatial distribution of bias respectively for the maximum and minimum values. This highlights that the raw output of the CMIP6 models fails to capture both extreme values and the temperature’s amplitude.

Raw simulations of precipitation (*pr*) presented an absolute relative bias of up to 60% and 40% for the long-term 90th percentile and maximum values, respectively (Fig. 4), corroborating with the findings of Pereima *et al*.^54^. Similar to *tasmax* and *tasmin*, the spatial distribution of the precipitation’s relative biases in the long-term extreme percentile was alike to those found for the long-term mean: dry biases in the Amazon and Pampa biomes and an overall wet bias in the rest of the country. The long-term maximum precipitation did not show a clear spatial pattern. Such as observed in the mean long-term, the climate models were not able to characterize the extreme values of *hur* and *sfcWind*. For the former, underestimations of more than 20% and 50% were respectively found for the 90th and 10th percentiles. For the latter, the errors were even more significant, with relative biases of around 80% and 100% for the 90th and 10th percentiles, respectively. Again, the simulations of *rss* exhibited the best performance with relative biases of nearly 10% of magnitude and a tendency to slightly underestimate the observations.

It is clear that the bias correction procedure significantly improved the estimations (Fig. 5), successfully removing most of the bias in both long-term mean and extreme values for almost all variables, except for the long-term maximum precipitation, where the improvement was not so perceptible. After the correction, the bias reduced to nearly 0% in all catchments (see histograms in Fig. 5). This is true even for *hur* and *sfcWind*, which were significantly misrepresented by the raw GCMs/ESMs. These results indicate that the QDM was able to overcome one of the main limitations of commonly used bias-correction methods: correcting systematic errors in different quantiles of the probability distributions of raw simulations, such as biases present in the mean and in the tail of the GCMs probability distribution^52^.

#### Seasonality

**Fig. 6:**
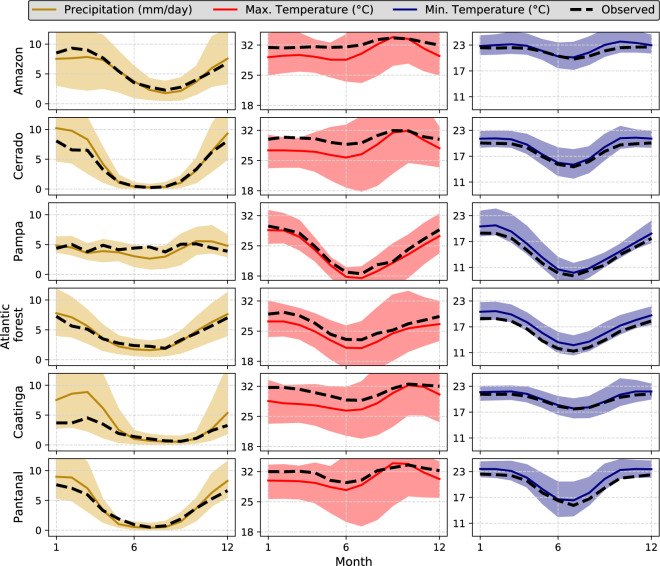
Long-term (1980–2013) monthly mean of precipitation and maximum and minimum temperature in each Brazilian biome. Highlighted lines represent the intra-annual cycle simulated by the raw *multi-model ensemble*. Dashed lines indicate the observed mean intra-annual cycle. Confidence intervals represent the maximum and minimum values simulated by the raw 19 CMIP6 GCMs/ESMs.

**Fig. 7:**
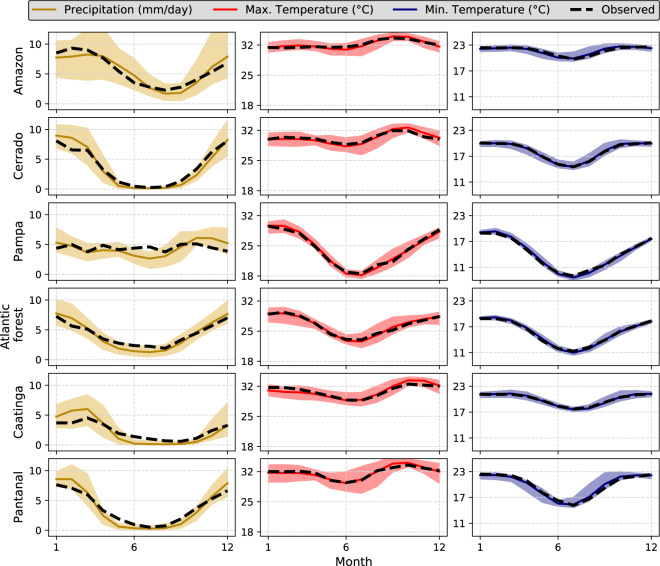
Long-term (1980–2013) monthly mean of precipitation and maximum and minimum temperature in each Brazilian biome. Highlighted lines represent the intra-annual cycle simulated by the bias-corrected *multi-model ensemble*. Dashed lines indicate the observed mean intra-annual cycle. Confidence intervals represent the maximum and minimum values simulated by the bias-corrected 19 CMIP6 GCMs/ESMs.

We assessed the intra-annual variability of *pr*, *tasmax*, and *tasmin* in each Brazilian biome (Figs. 6, 7 for raw and bias-corrected datasets, respectively). The intra-annual performance of the other variables is available in the accompanying Supplementary Material (Figures S1, S2 – Supplementary File 1) since these variables are not available in all 19 CMIP6 climate models. This investigation seeks to confirm if the simulations are able to reproduce the intra-annual cycle of the evaluated variables, an important aspect to assess especially for hydrological modelling purposes. Overall, both the raw and bias products exhibited good performance in reproducing the seasonal cycle of *pr*, *tasmax*, and *tasmin* (Figs. 6, 7). The confidence intervals, defined by the minimum and maximum values found in the 19 GCMs\ESMs, encompassed the observed pattern of the three variables in all biomes.

The smallest uncertainties, indicated by the confidence intervals, were found for *tasmin* followed by *pr* and *tasmax*. The models showed excellent performance in reproducing the intra-annual variability of *tasmin*, with a narrowed confidence interval and an ensemble mean close to the observations. Despite the large hydroclimatic variability in the country, we noted a clear seasonal pattern in *pr*. Both the raw and bias-corrected datasets exhibited a larger uncertainty in the characterization of the rainy season (October to March), as indicated by wider confidence intervals and a large difference between the ensemble mean and observations. Similar results were also found by Almazroui *et al*.^55^ when evaluating the performance of CMIP6 models in characterizing mean properties of rainfall and temperature in South America. In fact, more extreme rainfall events, which were significantly misrepresented by the models (Figs. 3, 4), are more likely to be experienced in the rainy season. In contrast, in the dry period, the ensemble mean approaches the observation showing also narrower confidence intervals. The exception here is the Pampa, where we did not find a clear pattern in both rainy and dry seasons since they are not well-defined in this biome^33^. An opposite situation was found for *tasmax*: larger confidence intervals in the dry period when the highest temperature amplitude and, consequently, climatic variability occur in Brazil^56^. In all biomes, raw models showed a slight underestimation of *tasmax*.

The bias-corrected simulations exhibited, again, excellent performance in characterizing the observed data (Fig. 7). They were able to significantly reduce the average biases approaching the observed and simulated monthly cycles, in addition to reducing the uncertainties expressed by narrower confidence intervals. Despite this, the limitations present in the raw simulations remained (Fig. 6), albeit with a less clear pattern: greater uncertainties in the simulation of *pr* and *tasmax* in the rainy and dry seasons, respectively. Similar conclusion can be drawn for *rss*, *hur*, and *sfcWind* (Figures S1, S2 - Supplementary File 1). Although not as significantly as observed for the *tasmax*, *tasmin* and *pr*, the bias correction improved the performance of the raw simulations in describing the monthly cycles of these variables, which showed smaller uncertainties and better accuracy. This can be explained by the fact that the GCMs/ESMs showed a lower performance in the characterization of the seasonality of these three variables (Figure S1 – Supplementary File 1), especially *sfcWind* and *hur*.

### Projected changes

#### Mean and extreme values

**Fig. 8:**
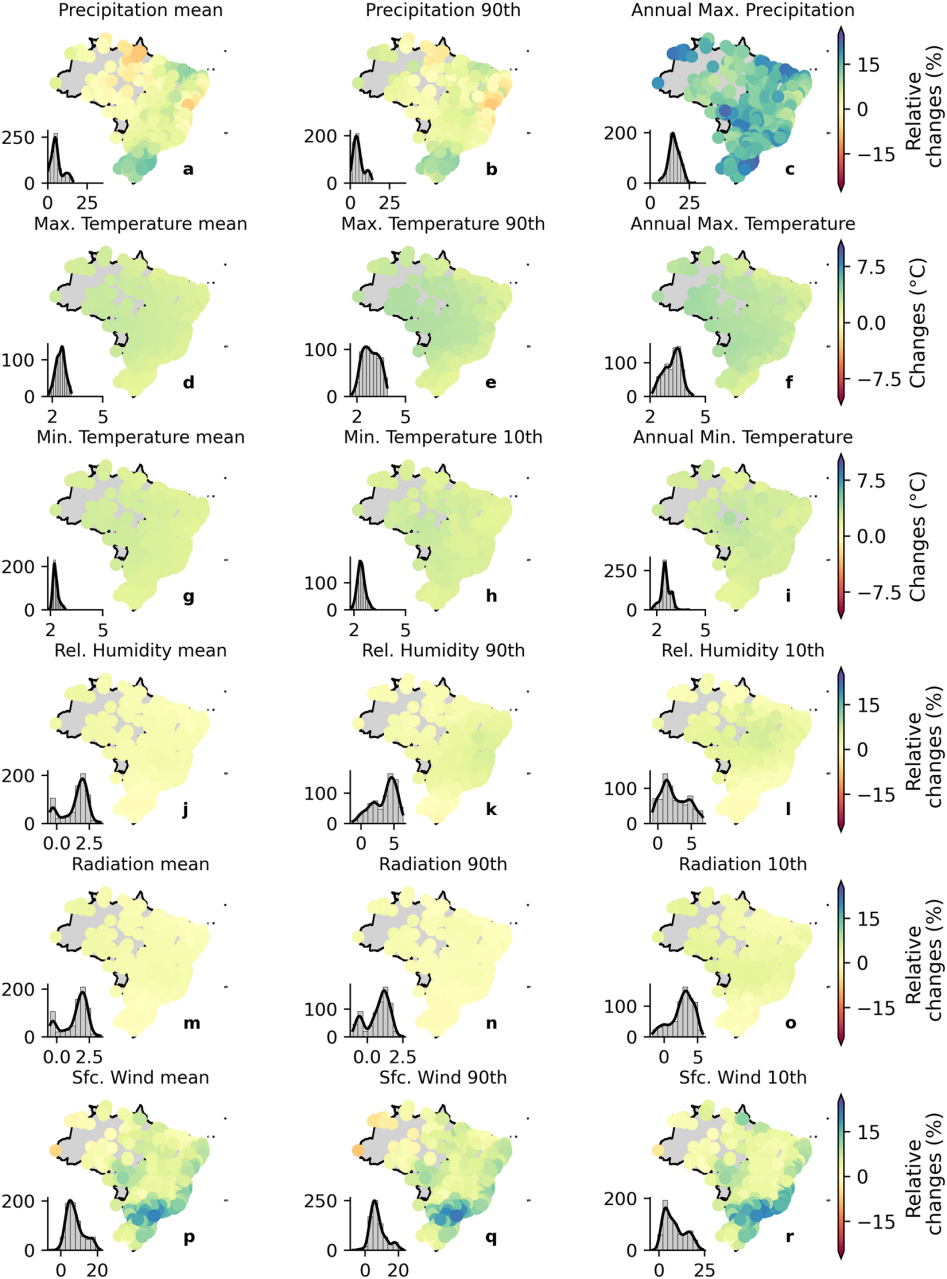
Relative changes in the long-term mean and extreme values of precipitation, maximum and minimum temperature, net shortwave solar radiation, relative humidity, and near surface wind speed between the historical (1980–2013) and distant future (2070–2100; SSP2-4.5) periods (bias-corrected catchment-scale dataset). Histograms in each panel indicate the frequency of occurrence of relative changes.

**Fig. 9:**
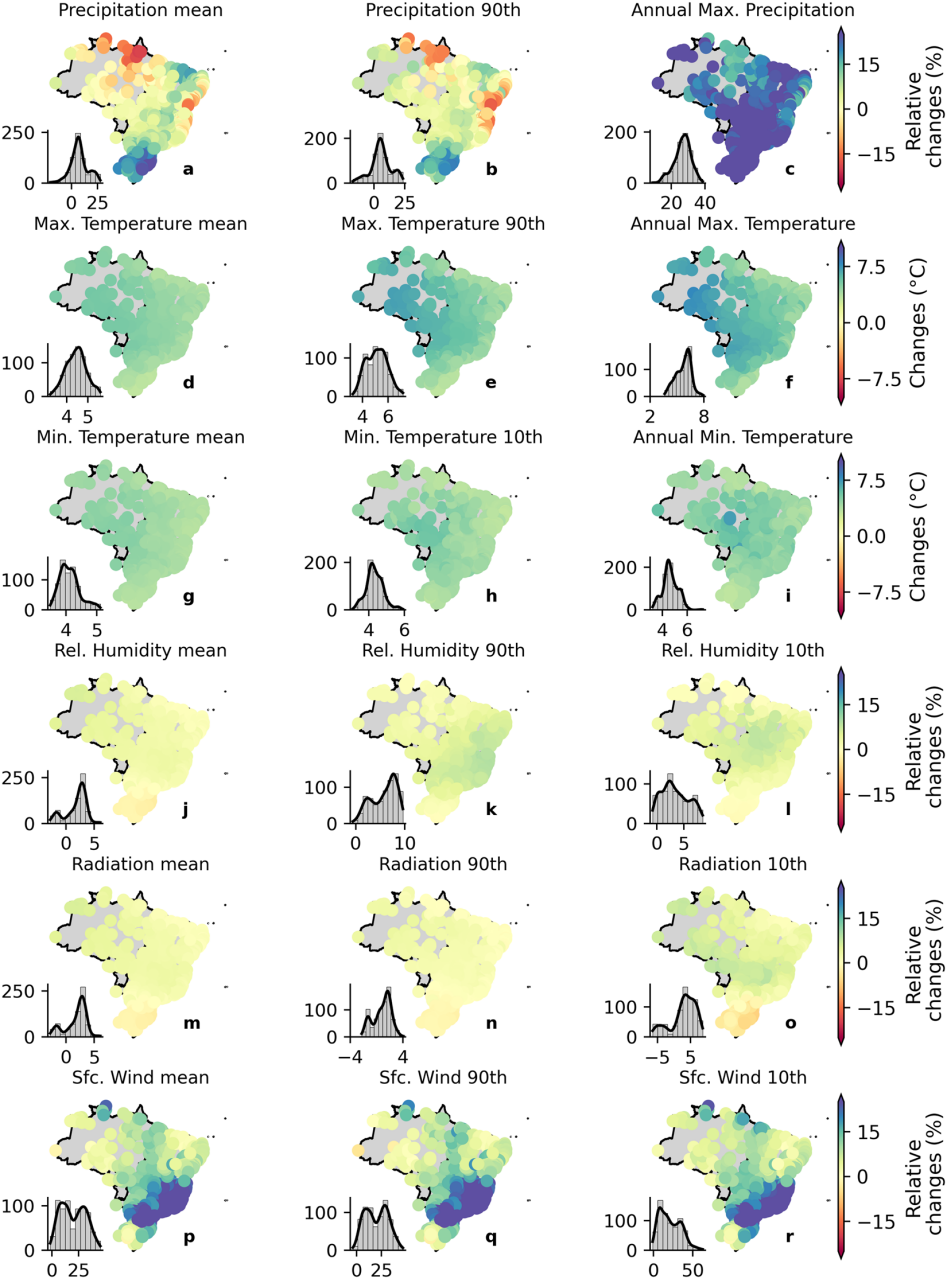
Relative changes in the long-term mean and extreme values of precipitation, maximum and minimum temperature, net shortwave solar radiation, relative humidity, and near surface wind speed between the historical period (1980–2013) and the distant future (2070–2100; SSP5-8.5) (bias-corrected catchment-scale dataset). Histograms in each of the panels indicate the frequency of occurrence of relative changes.

To analyze projected changes simulated by the bias-corrected CMIP6 models, we computed relative changes between the historical (1980–2013) and distant future (2070–2100) periods considering the long-term mean and extremes properties for both SSP2-4.5 and SSP5-8.5 scenarios (Figs. 8, 9, respectively). For *tasmax* and *tasmin*, we computed absolute changes (°C) to avoid extremely high values of relative changes due to denominator values close to zero. Projected changes simulated by the raw models are shown in the Supplementary Material (Figures S3, S4, respectively). It is worth noting that the projected changes were similar for both raw and corrected simulations (Fig. 8 and S3 for SSP2-4.5 and Fig. 9 and S4 for SSP5-8.5), indicating that the QDM method was capable to overcome another limitation of commonly used bias correction methods: it did not deteriorate trends and/or relative changes projected by the models, which may hamper the fully understanding of climate change effects^57,58^.

As expected, the changes simulated by the SSP5-8.5 scenario exhibited greater magnitude than those simulated by the SSP2-4.5. Among the six evaluated variables, *tasmax*, *tasmin*, *pr*, and *sfcWind* presented significant changes (>10%) between historical and future simulations in most parts of the Brazilian territory considering both scenarios. For *hur* and *rss*, the projected changes showed a smaller magnitude (<10%). Long-term mean precipitation and the 90th percentile exhibited similar spatial patterns of change over the country. A reduction in projected long-term mean *pr* was observed in the Amazon, Caatinga, and part of the Cerrado biomes. This expected reduction also corroborates the findings of Du *et al*.^59^. In the Pampa and Atlantic Forest biomes, a slight increase is expected. Similar conclusions can be drawn for the 90th percentile of precipitation. This pattern of change is more evident considering the SSP5-8.5 scenario (Fig. 9). Regarding the long-term maximum *pr*, a significant increase was observed (>10% for the SSP2-4.5 scenario and >20% for the SSP5-8.5 scenario) throughout the country, even in the biomes where a reduction in mean precipitation was projected (Figs. 8, 9).

In general, an increase in both *tasmax* and *tasmin* was observed in the two scenarios in Brazil. The projected increase in the long-term maximum temperature is slightly higher in the Amazon, Pantanal, and part of the Cerrado biomes than in the others. Furthermore, the positive magnitude of change in extreme values is larger than that projected for the long-term mean values. That is, both scenarios projected changes with greater magnitude in the extreme characteristics of *tasmax* (maximum and the 90th percentile) than in its mean values This is also valid for *tasmin*, but with a lower difference between magnitudes of change.

The projected changes in *rss* and *hur* showed smaller magnitude when compared with the other variables. For the former, an average increase in the long-term mean of about 2% and 4% is projected in the SSP2-4.5 and SSP5-8.5, respectively. Unlike *pr*, *tasmax*, and *tasmin*, there is no clear difference between the expected changes in mean and extreme values of *rss*, except the 10th percentile in the SSP5-8.5 scenario that shows a negative variation in the Pampa biome and in part of the Atlantic Forest biome and a positive variation in the central region of the country. The *hur* variable also showed a similar pattern of projected changes in the long-term mean and extreme values across the country, indicating a slight increase of <2.5% in the SSP2-4.5. For the SSP5-8.5 scenario, we noted slightly larger changes (<5%) in both the mean and extreme values. Interestingly, the 10th and 90th percentiles showed an opposite spatial distribution in the SSP5-8.5 scenario, in which we found larger increases in the 90th percentile and smaller increases in the 10th percentile of *hur*. Lastly, large increases in the *sfcWind* of 15% in SSP2-4.5 and 25% in SSP5-8.5 are expected especially in the Pampa (South region) and Atlantic Forest biomes (Southeast region). In the other regions, smaller increases were noted. It was also not possible to distinguish different spatial patterns between the changes in mean and extreme events.

#### Seasonality

**Fig. 10:**
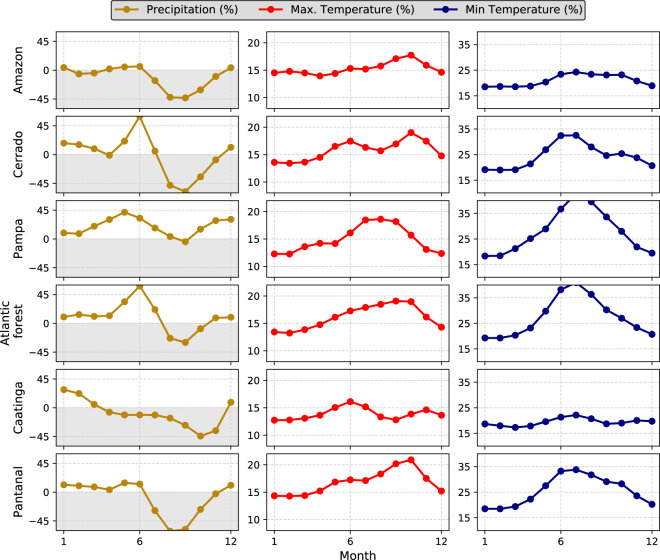
Relative changes in the long-term mean intra-annual cycles of precipitation and maximum and minimum temperatures between the historical (1980–2013) and distant future (2070–2100, SSP5-8.5) periods. Highlighted lines represent the changes in the intra-annual cycle simulated by the bias-corrected *multi-model ensemble*.

Significant changes in intra-annual cycles are also expected (Fig. 10). For better visualization, we only detailed the relative changes (*multi-model ensemble*) expected in the monthly averages of *pr*, *tasmax*, and *tasmin*. The results for the other three variables are provided in the Supplementary File 1 (Figure S5). We considered the bias-corrected simulations of the historical (1980–2013) and ‘distant’ future (SSP5-8.5; 2070–2100) to compute the relative changes. However, very similar changes were observed for the simulations without bias correction.

Changes in projected precipitation’ seasonal cycles varied among the biomes (Fig. 10). For the Atlantic Forest and Cerrado, a shift in seasonality is projected, indicated by a large increase in precipitation in the first months of the dry period (April to July) and a reduction in the last months (August and September). For the Amazon, Caatinga, and Pantanal biomes, an overall decrease in precipitation is expected, but with the maintenance of seasonal cycles. Finally, a general increase in precipitation is projected in the Pampas biome, however, with a heterogeneous distribution throughout the year: larger variations were observed at the beginning of both dry (April to June) and rainy seasons (October to December). We also found positive relative changes for *tasmin* and *tasmax*. For the former, a more significant increase is projected between May and September in all biomes, especially in the Pampas, Pantanal and Atlantic Forest biomes where the increase was more significant (up to 35%). The projected increase in *tasmax* is expected to be more uniform throughout the year than those projected for *tasmin*, being concentrated in the second half of the year, except for the Caatinga and Amazon biomes, which presented a similar rate of increase throughout the year for both *tasmin* and *tasmax*.

## Usage Notes

Here, we described the CLIMBra - Climate Change Dataset for Brazil, which provides raw and bias-corrected daily time series of six meteorological variables at both gridded and catchment scales using simulations of up to 19 CMIP6 GCM/ESMs. The simulations were provided for both historical (1980–2013) and future periods (2015–2100) forced by two different emission scenarios: SSP2-4.5 and SSP5-8.5. CLIMBra products may be useful for different hydroclimatic purposes such as hydrological modeling and climate change impact assessment. Moreover, CLIMBra may also be of interest to users not only in the hydrometeorological field but also in others such as agriculture, public health, and ecology. The high-resolution of the gridded data (0.25° × 0.25°) is key to developing regional assessments, providing information to decision and policy-making not only in Brazil but also in South America given the Brazil’s continental proportion and its role in global climate dynamics.

Our main product, the catchment-scale dataset, is provided as comma-separated values format (*.csv*), which is easier to handle and download in comparison with *netCDF* files. Both the gridded and catchment-scale datasets are freely available at the Science Data Bank (10.57760/sciencedb.02316). Despite the importance of the developed product, it is important to highlight some of its limitations. (1) Data users should be aware of significant bias when using our raw database due to its weak performance in representing observations, depending on its application. Thus, a performance analysis should be conducted to investigate whether the raw historical data are able to simulate observations. (2) Even exhibiting a better performance than the raw simulations (mainly in reproducing seasonal variability and extreme properties of the evaluated variables), the bias-corrected products may present inherent uncertainties, physically unrealistic values, and hide some fundamental deficiencies presented by the climate models. Finally, (3) data users should consider that the area-averaging process used to develop the catchment-scale dataset may hinder or smooth extreme events and misrepresent transboundary catchments.

## Supplementary information

Supplementary Material - File 1

## Data Availability

Pre- and post-processing tasks were carried out using the R-packages of the *Climate4R* project, extensively described in Bedia *et al*.^18^ and Iturbide *et al*.^49^. This framework was developed to address the needs of different climate-impact studies and includes a roll of R-packages to access, pre- and post-process, and visualize climate data. All the packages and documentation, including tutorials and example-notebooks, are available through the following Github link: https://github.com/SantanderMetGroup/climate4R.
