# Supplementary material for: CLIMBra - Climate Change Dataset for Brazil
Source: Sci Data. 2023 Jan 20;10:47. doi: 10.1038/s41597-023-01956-z (PMC9860025; doi:10.1038/s41597-023-01956-z)

Supplementary Material - File 1

CLIMBra - Climate Change Dataset for Brazil

André Simões Ballarin[1], Jullian Sousa Sone[1], Gabriela Chiquito Gesualdo[1], Dimaghi Schwamback[1], Alan Reis[1], André Almagro[2], and Edson Cesar Wendland[1].

Affiliations

1. Department of Hydraulics and Sanitation, São Carlos School of Engineering, University of São Paulo, CxP. 359, São Carlos, São Paulo, 13566-590, Brazil

2. Faculty of Engineering, Architecture and Urbanism, and Geography, Federal University of Mato Grosso Do Sul, CxP 549, Campo Grande, Mato Grosso Do Sul 79070-900, Brazil

Corresponding author(s): André S. Ballarin (andre.ballarin@usp.br)

Content: Figures S1 to S5

Figure S1. Long-term (1980-2013) monthly mean of relative humidity, solar net radiation, and near-surface wind speed, separated by Brazilian biomes. Highlighted lines represent the intra-annual cycle simulated by the raw 10 CMIP6-GCMs' *multi-model ensemble*. Dashed lines indicate the observed mean intra-annual cycle. Confidence intervals represent the maximum and minimum values simulated by the raw 10 CMIP6-GCMs.

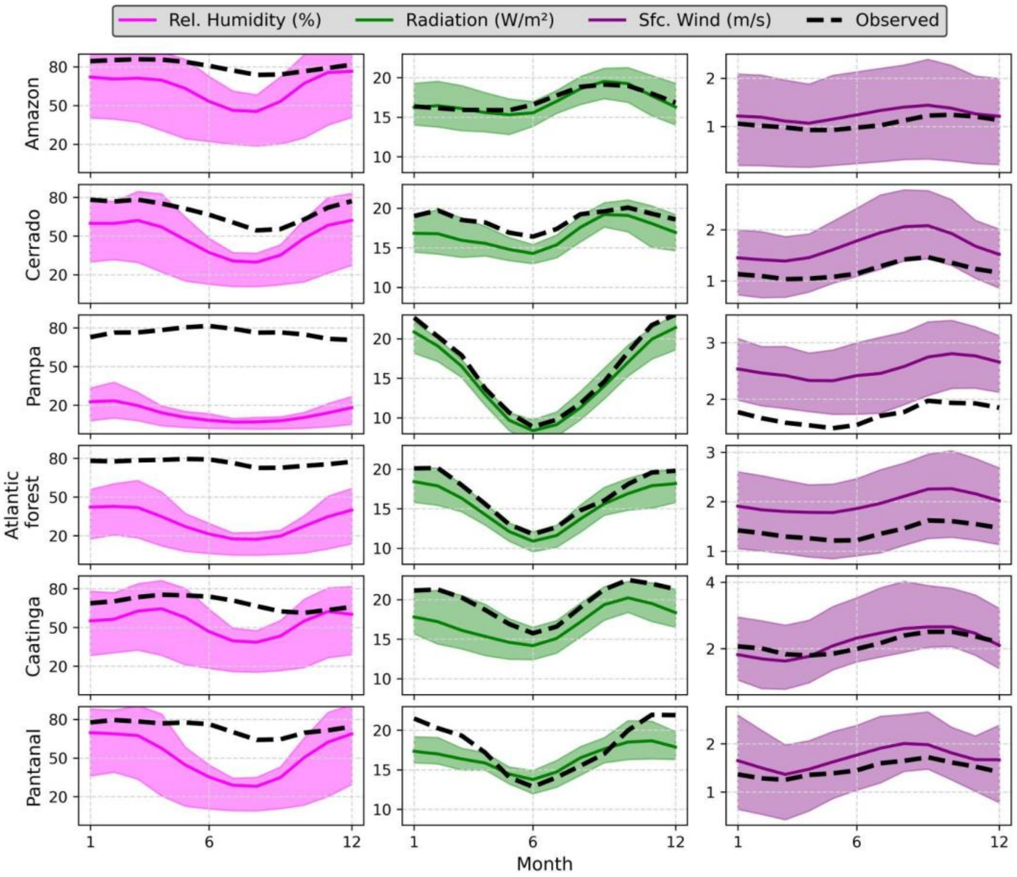

Figure S2. Long-term (1980-2013) monthly mean of relative humidity, solar net radiation, and near-surface wind speed. Highlighted lines represent the intra-annual cycle simulated by the bias-corrected 10 CMIP6-GCMs' *multi-model ensemble*. Dashed lines indicate the observed mean intra-annual cycle. Confidence intervals represent the maximum and minimum values simulated by the bias-corrected 10 CMIP6-GCMs.

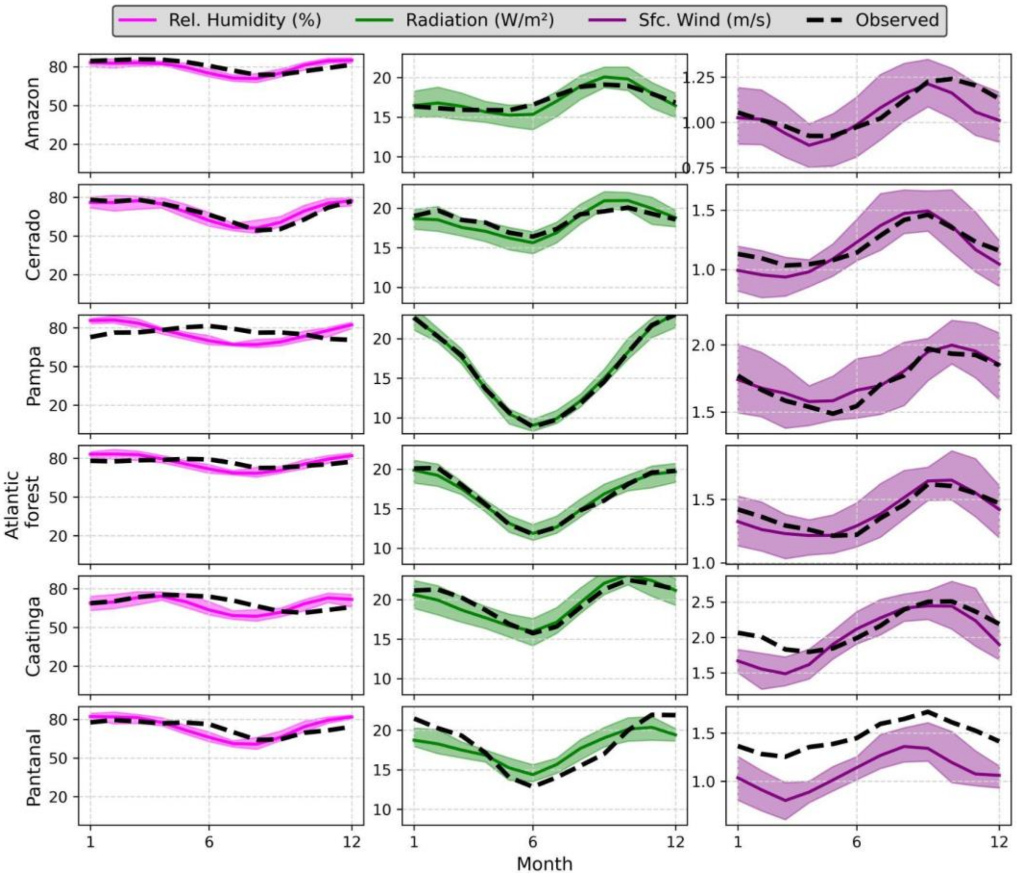

Figure S3. Relative changes in the long-term mean and extreme values of precipitation, maximum and minimum temperature, net shortwave solar radiation, relative humidity, and near-surface wind speed between the historical (1980-2013) and distant future (2070-2100; SSP2-4.5) periods (raw catchment-scale dataset). Histograms in each panel indicate the frequency of occurrence of relative changes.

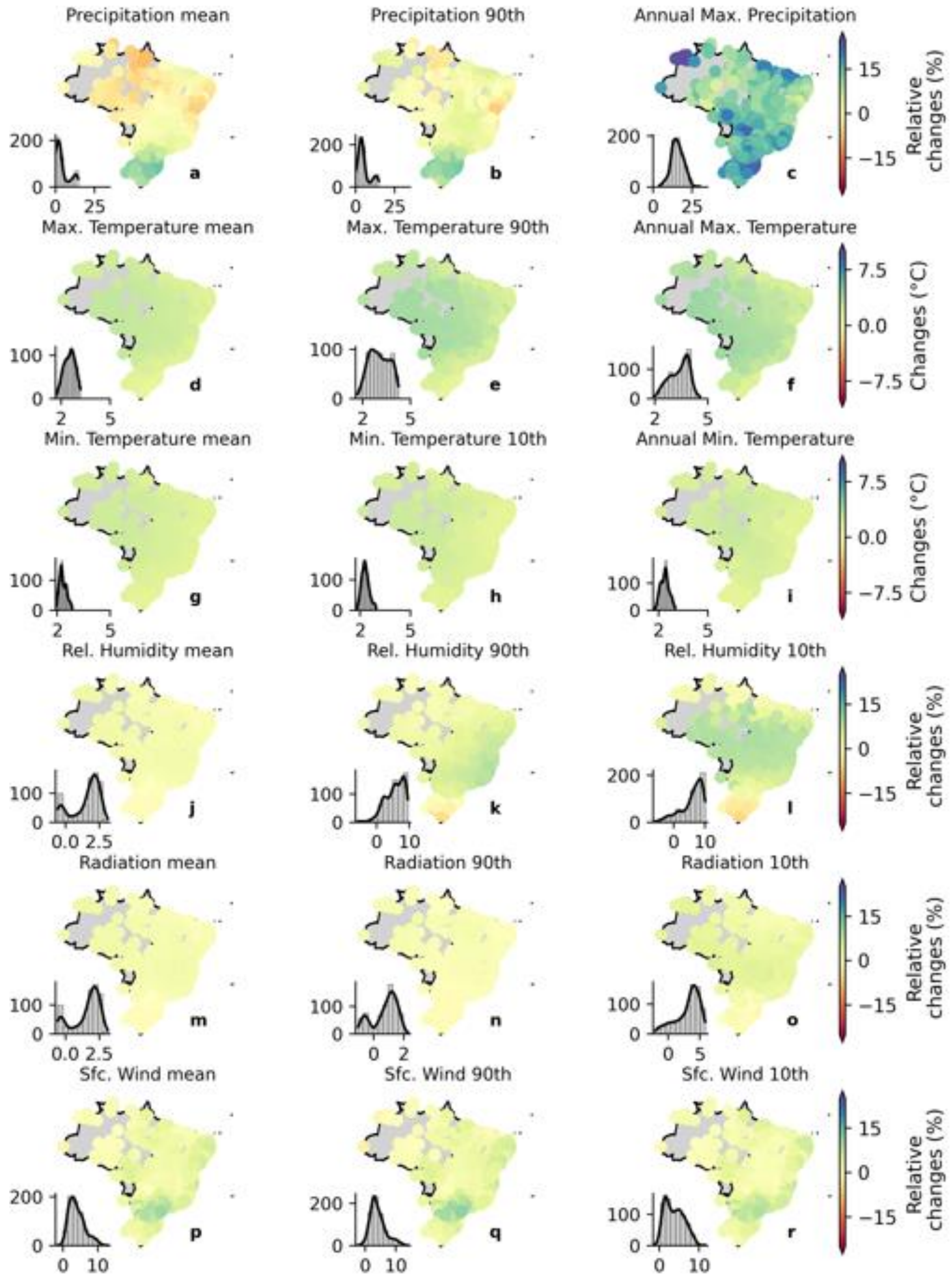

Figure S4. Relative changes in the long-term mean and extreme values of precipitation, maximum and minimum temperature, net shortwave solar radiation, relative humidity, and near-surface wind speed between the historical (1980-2013) and distant future (2070-2100; SSP5-8.5) periods (raw catchment-scale dataset). Histograms in each panel indicate the frequency of occurrence of relative changes.

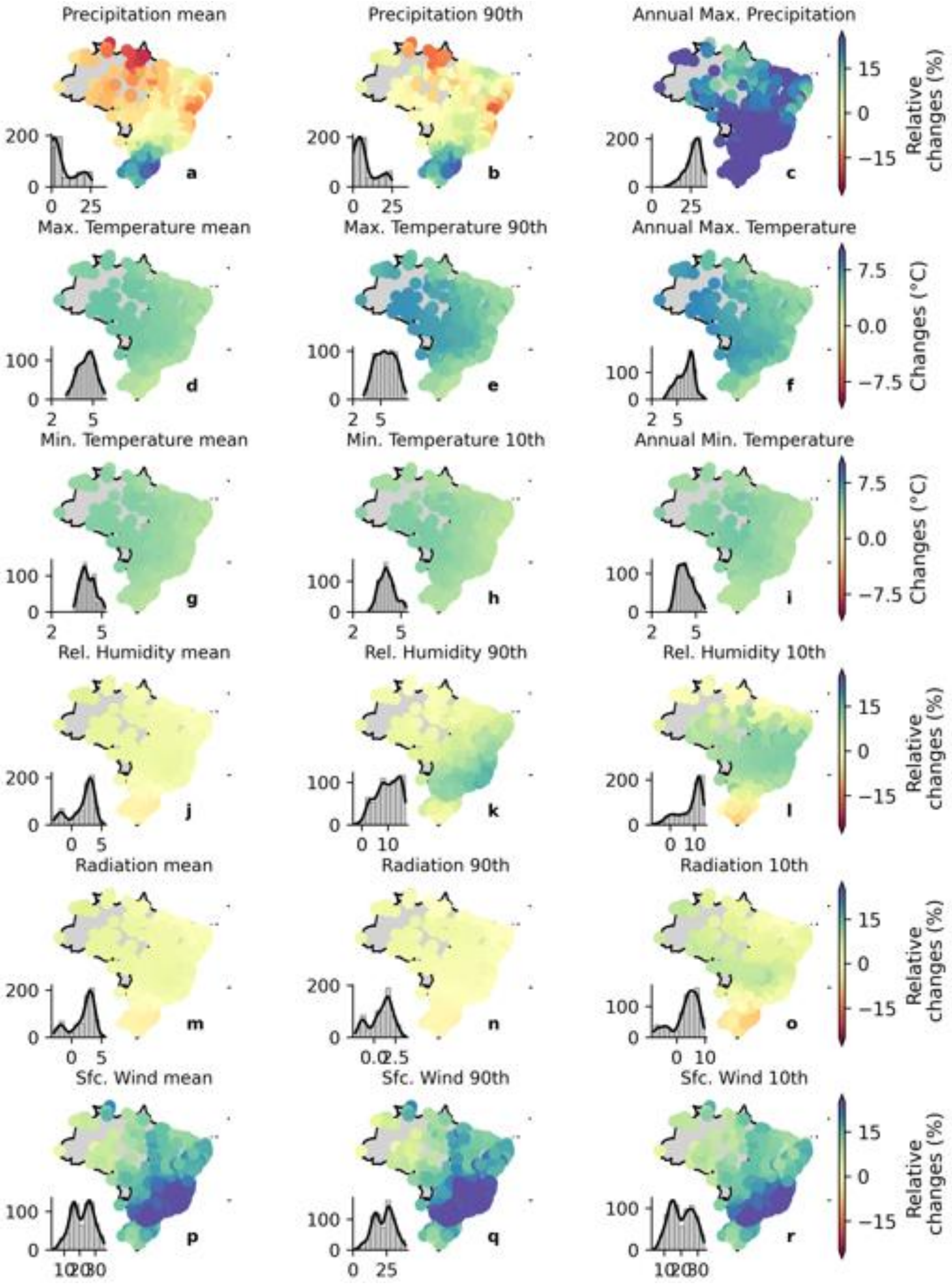

Figure S5. Relative changes between the historical (1980-2013) and distant future (2070-2100, SSP5-8.5) periods in the long-term mean intra-annual cycles relative humidity, solar net radiation, and near-surface wind speed, separated by biomes. Highlighted lines represent the changes in the intra-annual cycle simulated by the bias-corrected 10 CMIP6-GCMs' *multi-model ensemble*.

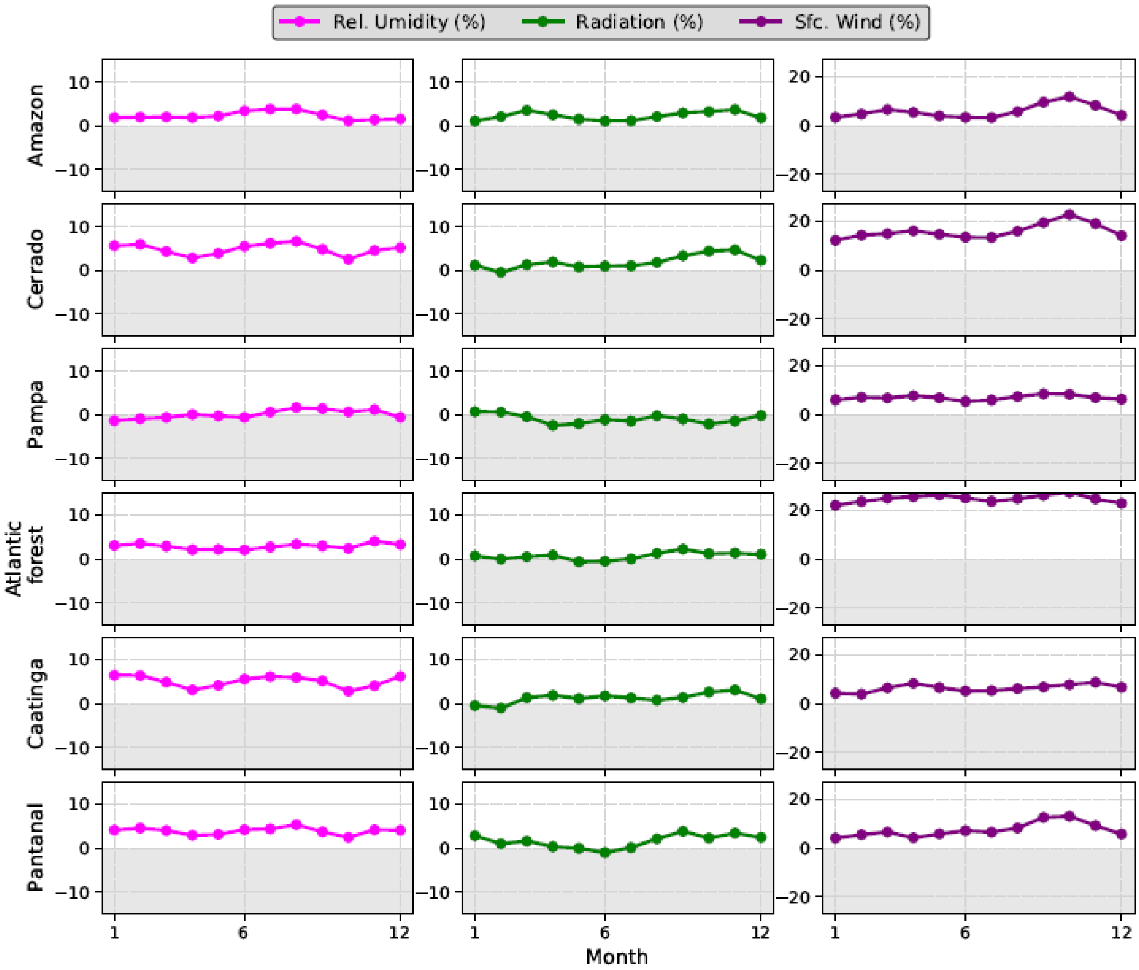

Supplement: Supplementary file 1 — Supplementary Material - File 1 [file 41597_2023_1956_MOESM1_ESM.pdf]
